# Shear Behaviors of RC Beams Externally Strengthened with Engineered Cementitious Composite Layers

**DOI:** 10.3390/ma12132163

**Published:** 2019-07-05

**Authors:** Guan Wang, Caiqian Yang, Yong Pan, Fawang Zhu, Kai Jin, Kefeng Li, Antonio Nanni

**Affiliations:** 1 Key Laboratory of Concrete & Pre-stressed Concrete Structures of the Ministry of Education, Southeast University, Nanjing 211189, China; 2 Department of Civil, Architectural, and Environmental Engineering, University of Miami, Miami, FL 33146, USA; 3 College of Civil Engineering and Mechanics, Xiangtan University, Xiangtan 411105, China; 4 Jiangsu Eastern Expressway Management Co., Ltd., Yancheng 224000, China

**Keywords:** shear, ECC, FBG sensor, truss and arch model, strengthening effect

## Abstract

The shear behaviors of reinforced concrete (RC) beams externally strengthened with engineered cementitious composite (ECC) layers were studied and the strengthening effect was evaluated based on a truss and arch model. The beams were designed without web reinforcement in the middle part and ECC was sprayed onto both sides of the beams to the designed thicknesses, which were 20 mm and 40 mm. A series of four-point bending experiments were conducted and analyzed. The development of the shear strain in each side of the beams was recorded by strain rosettes formed with three fiber Bragg grating (FBG) sensors. The thickness of ECC layers, reinforcement ratios, and shear span-to-depth ratios were considered and analyzed. This is an effective way to shear strengthen RC beams with ECC layers. The ultimate load of the strengthened specimen can be improved by 89% over the control specimen. Strengthening an RC beam into an under-reinforced beam should be avoided. The FBG sensors are suitable to measure and monitor the development of shear strain in the side of the strengthened specimen. Based on the truss and arch model, an evaluation of the shear strengthening effect was established and the results agree well with the experimental results.

## 1. Introduction

Engineered cementitious composite (ECC) is a class of ultra-ductile fiber reinforced cementitious composite invented in the early 1990s [1] with a design based on micro-mechanics [2]. Its tensile ductility, with no more than 2% volume fraction of polyvinyl alcohol (PVA) or polypropylene fibers, can reach more than 3% with multiple cracks distributing in the interface [3,4,5]. Many studies have been performed to emphasize that ECC has many great properties such as ultra-high tensile strength, strain hardening behavior, stable cracking, excellent impact resistance, and good energy dissipation capacity [5,6,7,8,9,10]. 

The shear behaviors of ECC material and structure have been studied and the results demonstrate that the pseudo-strain-hardening tensile properties of ECC materials can be successfully translated into an advantageous structural shear response [11,12]. It was found that the ECC significantly enhanced the shear behavior of the short span beam under cyclic loading [13]. Further, the shear strength of specimens increased with the volume percentages of PVA fiber [14]. The structural behaviors of steel reinforced ultra-high performance ECC beams under bending were experimentally explored and the results of the test indicated the feasibility of utilizing their ultra-high performance to substantially reduce or replace the steel bar in structural members [15]. Conforti and Minelli conducted research on the numerical modeling of the shear behavior of deep beams with fibers or no shear reinforcement in order to study the size effect influence on the shear behavior of fiber reinforced concrete (FRC) elements [16]. Moreover, a material-performance-based database for FRC and reinforced concrete (RC) was conducted which discussed the influence of the different factors affecting the shear strength both in FRC and RC samples [17]. All the parameters involved in shear have been studied and have great reference value for shear research.

Due to the excellent performance, especially the ultra-high tensile strength and strain hardening behavior, and the cost-effective development of ECC, it has been regarded as a suitable material for strengthening RC structures [6,18,19,20,21,22]. Kim et al. studied crack damage mitigation and the shear behavior of shear-dominant RC beams repaired with ECC. The experimental results show that the use of an ECC layer leads to a substantial increase in the shear strength and ductility of the RC beams after the peak load. The results also indicate that ECC layers can be effective repair material for enhancing the control of cracking to help protect the concrete from the migration of aggressive agents in severe environments [23]. Research on the mechanical behavior of steel reinforced ECC or ECC/concrete composite beams under reversed cyclic loading was conducted and the steel reinforced ECC beams showed better seismic performance in terms of load carrying capacity. Beams failed in shear showed a more significant improvement than those failed in flexure [24]. Afefy and Mohamed placed pre-cast and cured ECC strips in the tension cover zone of one-way reinforced RC slabs beside the main steel reinforcement. Test results showed that the ECC strips enhanced the structural performance of the slabs at both service and ultimate limit states [25]. Jiang et al. found that introducing a new ECC layer on the tensile side improves the cracking control and flexural behavior of a fiber-reinforced polymer (FRP) reinforced sea sand and seawater concrete beam, especially in the serviceability limit state [26]. Hung and Chen conducted research on ECC jacketing for retrofitting shear-deficient RC members. They found that the ECC jacket without steel meshes can improve the cyclic behavior of the original element considerably. The behavior of the retrofitted beam at the performance level of the ultimate limit state can be further enhanced by reinforcing the ECC jacket with a steel mesh [27].

With the development of sensing technology, several new measures have been developed, such as the digital image correlation technique [26], microwave near-field detecting technique [28], infrared thermography technique [29], and fiber Bragg grating (FBG) sensor measurement technique [30,31]. Due to the large measurement range, high-precision, stability, and measurable for crossing crack within the range, the FBG sensor is suitable for use in the field of civil engineering, especially for the measurement of the tensile strain of FRP, steel, concrete, and fiber reinforced concrete etc.

For the calculation method of RC beams’ shear bearing capacity, Ritter [32] and Morsch [33] published their papers independently. In their studies, they presented the truss Model—based on which the shear bearing capacity of an RC beam can be simplified and calculated as if it was a truss. The arch action in RC members subjected to shear force has been recognized by many researchers [34,35,36,37]. Thus, the truss and arch model has been developed and accepted by many national codes and widely used for structural design and evaluation. Both the Truss and Arch actions contribute to the shear capacity of RC beams [38,39,40,41,42].

In this paper, all the specimens described are without web reinforcement in the middle part. The experiments were conducted to study the shear strengthening effect of ECC layers. Four-point bending experiments for control specimens as well as specimens strengthened with 20 and 40 mm ECC layers at both sides were conducted and analyzed. Three linear variable differential transformers (LVDTs) were placed at the mid-span and two loading points, respectively. Two rosettes which formed with three FBG sensors were applied to measure the shear strain on the side of each beam. The mid-span load-deflection response and shear strain development on the sides of the beams were recorded and compared. The main test parameters included reinforcement ratio, the thickness of the ECC layers, and shear span-to-depth ratio.

## 2. ECC Material Properties

### 2.1. Compressive Strength

The ECC material was prepared by the procedure suggested by Zhou [43] and the volume fraction of PVA fiber was 1.5%. Three cubes with a size of 100 mm were used to conduct compressive tests at the curing age of 28 days. The specimens were fabricated and cured according to the standard [44]. Then the cubes were loaded with an MTS hydraulic servo loading system (MTS-1000 kN, Eden Prairie, MN, USA). The average value of the compressive strength is 55.9 MPa (4.20%, Covariance).

### 2.2. Direct Tensile Strength

**Figure 1:**
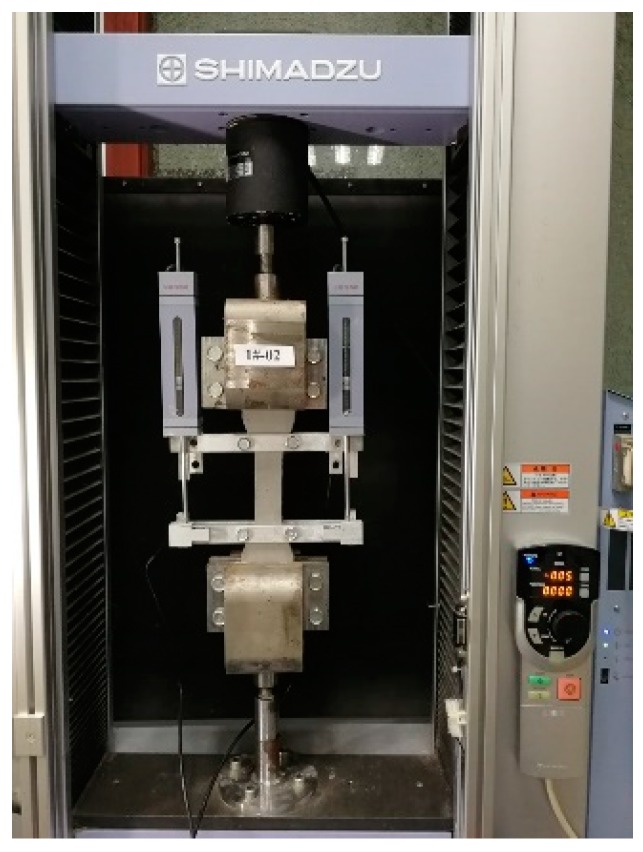
Direct tensile test setup.

Three dog-bone specimens were used to conduct direct tensile tests for ECC. The specimen and test setup are shown in Figure 1. The middle part of the specimen has a thickness of 13 mm, a width of 30 mm and a length of 80 mm. The direct tensile tests were conducted at a speed of 0.2 mm/min using Shimadzu AG-Xplus-10 kN (Kyoto, Japan). As shown in Figure 1, the head and bottom of a specimen were fixed to the loading frame and two LVDTs were used to record the deflection of the middle part of the specimens. 

**Figure 2:**
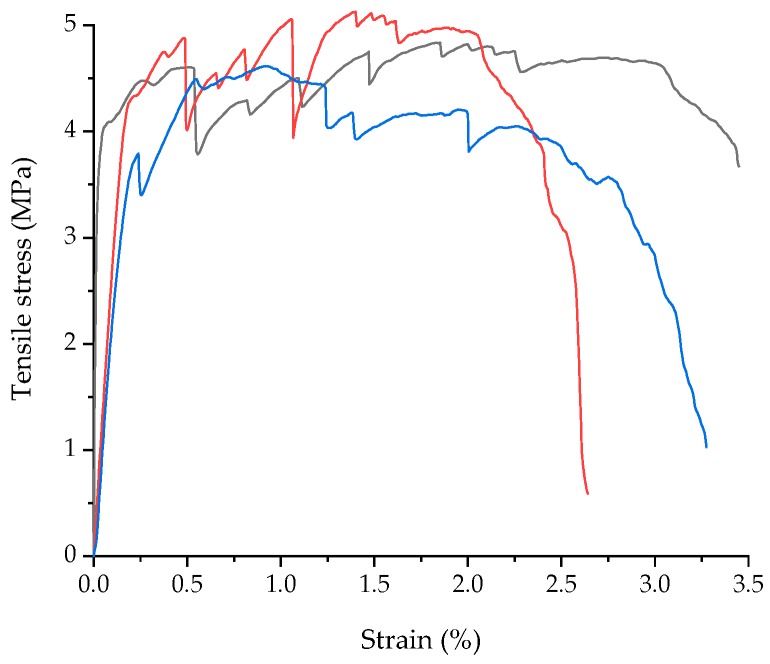
Tensile stress-strain curves of ECC.

The tensile stress-strain curves of ECC are shown in Figure 2. The tensile strain curves show that ECC has a remarkable tensile strain hardening behavior. Different from common concrete with a brittle failure mode, the ECC specimen can reach high tensile strain after the first crack. The cracking tensile strength is 4.05 MPa (6.67%, Covariance), the maximum tensile strength is 4.84 MPa (5.28%, Covariance).

## 3. Four-Point Bending Experiments

### 3.1. Materials Properties and the Fabrication of Specimens

The properties of concrete, ECC and steel rebars used in the experiments are shown in Table 1. In Table 1, “Dia” means the diameter of rebar, “Net Area” means the cross section area of the rebar, “*f_c_*” means the cube compressive strength of concrete and ECC, “*f_t_*” means the tensile strength of concrete and ECC, “*f_y_*” means the yield strength of rebar, “*ε_y_*” means yield strain of rebar, “*f_u_*” means the ultimate strength of rebar, “*ε_u_*” means the ultimate strain, and “*E*” means the elastic modulus. 

Commercial concrete was poured into wood molds, where the steel bars were already located according to the design. A point vibrator was used to facilitate compaction. The demoulding for the concrete beams was conducted after 10 days. The beams were designed without web reinforcement in the middle parts. Only four steel stirrups with a diameter of 10 mm were placed at each end of the beams with the spacing of 50 mm to avoid fracture happening caused by stress concentration at the supports.

In the experiment, sprayed ECC layers with a thickness of 20 and 40 mm were used to strengthen the RC beams. Based on the research conducted by Kim et al., the sprayed ECC exhibits strain-hardening behavior with strain capacities comparable with the cast ECC with the same mixture proportion [45].

The procedure consists of the following steps: (1) Chisel both sides of each beam, then clean and wet the sides to a standard dry surface condition. (2) ECC was fabricated according to the procedure suggested by Zhou [43]. (3) An interface agent (constituents: 0.85 cement + 0.05 silica fume + 0.1 expansive agent + 0.3 water, by mass) was applied to the sides of the beams. (4) ECC was sprayed onto the sides of the beams to the designed thickness. (5) The strengthened beams were covered with a polyethylene sheet to prevent the loss of moisture and cured for 28 days at room temperature. During the curing, water was sprayed onto the surfaces of the ECC layers to make sure that the hydration could process well.

### 3.2. Experimental Setup and Instruments

**Figure 3:**
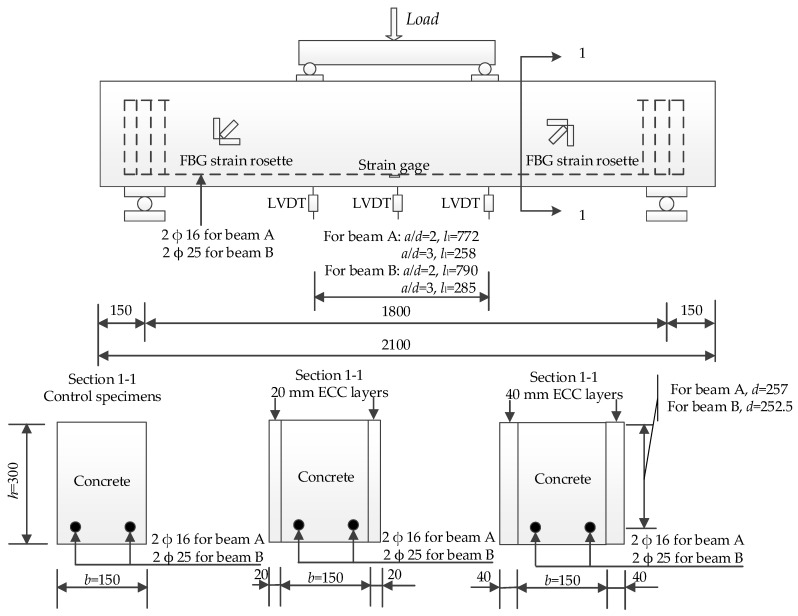
Sketch of the specimens (mm).

The length (*L*) for all specimens is 2100 mm. And all specimens were simply supported with a span (*l*) of 1800 mm. The sketch of loading setup and distribution of strain sensors in specimens is shown in Figure 3. The details of the specimens are listed in Table 2. Type A beam is that one strengthened with two longitudinal reinforcements with a diameter of 16 mm. And type B beam is that one strengthened with two longitudinal reinforcements with a diameter of 25 mm. The specimen ID of control specimens consists of two parts connect with “-”. The first part is composed of two letters and the first one is always “C” which means “Control”, and the second one is “A” or “B” which stands for the type of the beam. The number behind “-” means the span-to-depth ratio (*a*/*d*) which represents 2 or 3. For the strengthened specimens, the naming rule is similar to that of control specimens but the letter “S” means “strengthened” and the number between the two “-” means the thickness (*t*) of strengthened ECC layer of one side. For type A beams, the loading spans (*l_l_*)were 772 mm for the specimens whose *a*/*d* represent 2.0, and 258 mm for the specimens whose *a*/*d* represent 3.0. For type B beam, the *l_l_* was 790 mm for the specimens whose *a*/*d* represent 2.0, and 285 mm for the specimens whose *a*/*d* represent 3.0. The cross-section (*b* × *h*), reinforcement ratios (*ρ*) before and after the strengthening treatment are also listed. The specimens were loaded until final failure with an MTS hydraulic servo loading system (MTS-1000 kN) under a load control manner.

In this test, FBG sensors with a gauge length of 15 cm were used to measure the shear strain. There were two strain rosettes on the side of each specimen to measure the development of shear strain, and each strain rosette was formed with three FBG sensors. There was one strain gage stick on each one of the main reinforcements at the middle location. Three LVDTs were used to measure the deflections at the mid-span and the two loading points. The initiation and propagation of cracks in the concrete were observed during the experiments. 

### 3.3. Experimental Results and Discussion

#### 3.3.1. Behaviors between Load and Mid-Span Deflection

**Figure 4:**
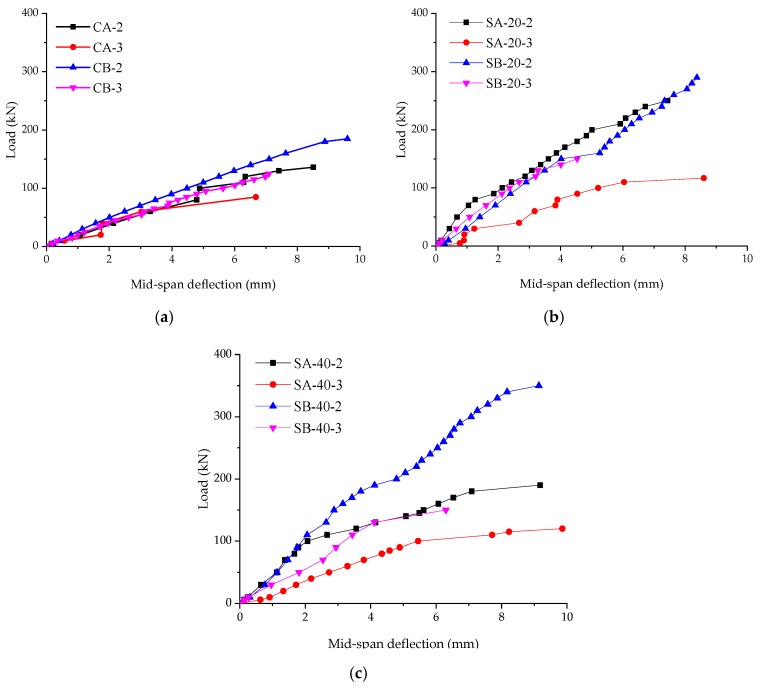
Load and mid-span deflection curves. (**a**) Control specimens; (**b**) Specimens strengthened with 20 mm ECC layers; (**c**) Specimens strengthened with 40 mm ECC layers.

The load and mid-span deflection curves of the specimens are shown in Figure 4. The results are demonstrated in Table 3. In Table 3, *P_u_* means the ultimate load of the specimen, *y* means the ultimate deflection at the mid-span, Δ*P*_u_ means the increment of ultimate load of the strengthened specimen compares with the corresponding control specimen, *S* means the increment percentage of ultimate load, *τ* means the average shear stress in a section of the parts between a support point to the closed loading point under the ultimate load. For control specimens, the load and mid-span deflection curves can be found in Figure 4a. With an increase in load, the deflection of the specimens increases linearly until failure. No yielding stage was observed for any of the control specimens.

For specimens strengthened with 20 mm ECC layers, the load and mid-span deflection curves can be found in Figure 4b. With increase in load, the deflections increase linearly until the failure happened. Compare with the control specimens, the ultimate loads and mid-span deflections are larger. The slope of the curve which represents the specimen SA-20-3 is smaller than that of the other three curves.

For specimens strengthened with 40 mm ECC layers, the load and mid-span deflection curves can be found in Figure 4c. For SA-40-2, the ultimate load is even lower than that of SA-20-2. And the ultimate load of SA-40-3 is almost the same with that of SA-20-3. It can be noticed from Table 2 that the *ρ* of the specimens SA-40-2 and SA-40-3 is 0.58%. According to the code for design of concrete structures, the minimum limit reinforcement ratio should be the larger one between 0.20% and 45*f_t_*/*f_y_*% (0.65%) [44]. So, SA-40-2 and SA-40-3 are under-reinforced beams. The dowel action of reinforcing bars is not enough, so the materials’ performances of concrete and ECC have not been fully used. It should avoid strengthening a beam to an under-reinforced one. For SB-40-2, the ultimate load increased by about 21% compared with the specimen SB-20-2. However, for SB-40-3, the ultimate load is almost the same as that of SB-20-3. This means when *a*/*d* is 3, the increment of the shear bearing capacity of the specimen is not sensitive to the increment of the thickness of ECC layers. 

According to the curves, the conclusion can be drawn that it is an effective way for strengthening an RC beam with ECC layers. However, the thicker ECC layers are not always the better. Strengthening a beam to an under-reinforced beam should be avoided.

#### 3.3.2. Shear Strains and Stress of the Specimens

**Figure 5:**
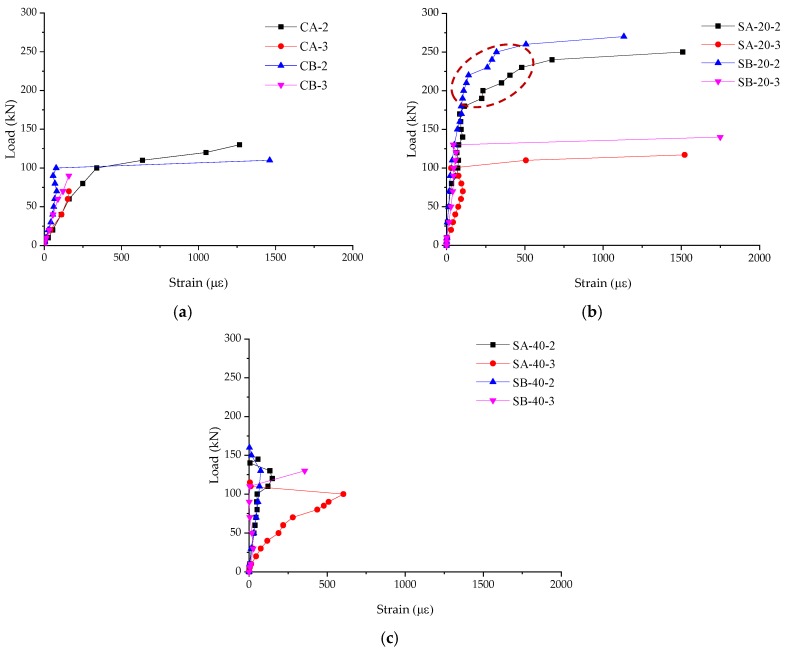
Shear strains in the side of the specimens. (**a**) Control specimens; (**b**) Specimens strengthened with 20 mm ECC layers; (**c**) Specimens strengthened with 40 mm ECC layers.

Figure 5 shows the shear strains in the side of the specimens. Figure 5a shows the shear strains of the control specimens. The shear strains increase linearly with the increase in loads, and then the strains increase greatly. The failure happened once the main cracks initiated and formed.

Figure 5b shows the shear strains in the side of the specimens strengthened with 20 mm ECC layers. For SA-20-3 and SB-20-3, the shear strains increase linearly with the increase in loads, then the shear strains increase greatly when the main cracks formed with failures happened. However, for SA-20-2 and SB-20-2, the shear strains increase slowly with an increase in load at the beginning stage, then the rates of strain growth increase (as shown in the red circle of Figure 5b). After that, the shear strains increase greatly with the main cracks formed and failures happened. The stages inside the red circle are kind of “yielding stages”.

Figure 5c shows the shear strains in the side of the specimens strengthened with 40 mm ECC layers. With the increase in loads, the shear strains increase. For the specimens SA-40-2, SA-40-3, and SB-40-2, the shear strains decrease sharply before failure happened. This is because the shear strains in the sides of the specimens released as the debonding happened. For specimen SB-40-3, a visible big crack formed within the range of FBG sensor at the end stage. Overall, the shear strains of the specimens are quite lower, and the regularity of the curves is not as good as that of the control specimens or the specimens strengthened with 20 mm ECC layers. From the curves, the shear failures of the specimens happened in brittle modes, but for SA-20-2 and SB-20-2, “yielding stages” were captured by FBG sensors. It is useful to shear strengthen RC beams with ECC layers. And FBG sensors can be used to captured and monitor the development of shear strains of the specimens.

**Figure 6:**
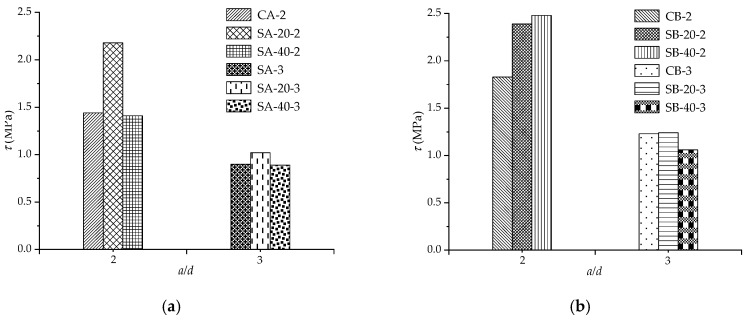
Ultimate shear stress of the specimens. (**a**) Type A specimens; (**b**) Type B specimens.

Figure 6 shows the ultimate shear stress of the specimens. Figure 6a shows the shear stress of type A specimens. Figure 6b shows the shear stress of type B specimens which is higher than that of the corresponding type A specimen due to higher reinforcement ratio. From Figure 6a, *τ* of SA-20-2 is 51.9% higher than that of CA-2. The strengthening of 20 mm ECC layers lead to the specimen reaches a higher load and deflection contribute to that. However, the shear stress of SA-40-2 stays at the same level as CA-2 due to the over strengthening of 40 mm ECC layers. When the *a*/*d* is 3, the shear stress stays at the same level no matter for type A or B specimens due to the ultimate loads are close to each other. For the type B specimens, the *τ* increase with the increase of the thickness of ECC layers when *a*/*d* represents 2. The strengthening effect of ECC layers is reflected.

#### 3.3.3. Failure Modes of the Specimens

**Figure 7:**
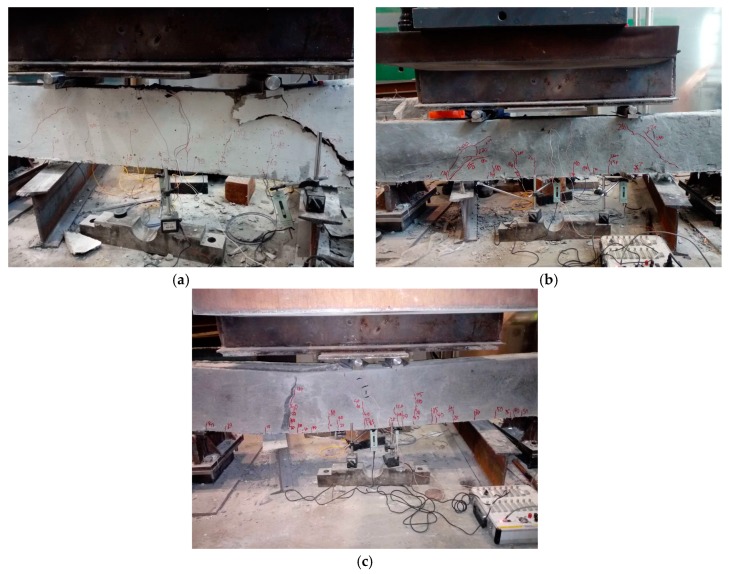
Typical failure modes of the specimens. (**a**) control specimen; (**b**) specimen strengthened with 20 mm ECC layers; (**c**) Specimen strengthened with 40 mm ECC layers.

The typical failure modes of the specimens are shown in Figure 7. For control specimens, the typical failure mode can be found in Figure 7a. All control specimens showed typical brittle failure. With increase in loads, the hair cracks initiated on the sides in the flexural section and the failure happened once the main cracks formed which located at the loading points. No yielding stage happened in any of the control specimens.

For specimens strengthened with 20 mm ECC layers, the typical failure mode can be found in Figure 7b. All the specimens showed shear failure. Compare with the control specimens, there are more cracks distributed in the strengthened specimens with narrower space. For SA-20-2 and SB-20-3, the ECC layers peeled off from the beams after the failures happened. For SA-20-3, one main crack formed in the ECC layer at the loading point. For SB-20-2, there is no debonding phenomenon happened and the quantity of cracks distributed in SB-20-2 is more than the others.

For specimens strengthened with 40 mm ECC layers, the typical failure mode can be found in Figure 7c. The debonding failure happened to all the specimens strengthened with 40 mm ECC layers. Before debonding failures happened, the cracks initiated and developed with the increase in load, then partly debonding happened, after that, few cracks initiated and developed in the ECC layers. 

## 4. Evaluation for the Strengthening Effect

### 4.1. Truss Model

For web reinforced RC beams, stirrups can be regarded as tension members, and concrete between cracks can be regarded as compressive members. Because of high tensile strength, high tensile strain, and strain hardening behaviors of ECC, in this paper, the tensile behaviors of ECC are regarded as the tension member. Further, the compressive behaviors of ECC between cracks work as compressive members in the truss model. 

**Figure 8:**
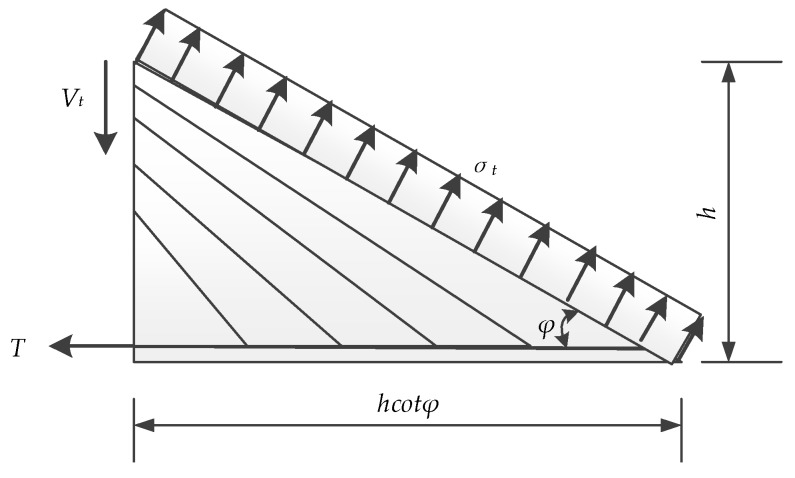
Left isolation body for Truss Model.

Based on the equilibrium condition of the left isolation body which is shown in Figure 8, the bearing capacity of the truss can be calculated by the Equation:

(1) $$V_{t}=\eta \beta \sigma_{t}\frac{h}{\cos\varphi }t\cos\varphi$$

In Equation (1), *η* is the reduction coefficient which is related to the bonding behaviors of the ECC layers and concrete beam. The factor *β* is used to show the reinforcement ratios’ effect on the ultimate loads of the specimens. The reinforcement ratios were changed as ECC layers applied onto the sides of the beams. *σ_t_* is the tensile strength of ECC. *h* is the height and *t* is the thickness of ECC layers.

**Figure 9:**
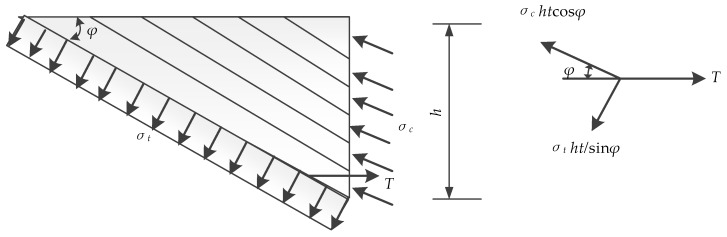
Right isolation body for Truss Model.

Take the right isolation body to study, which is shown in Figure 9. The vertical equilibrium condition is shown in the Equation (2):

(2) $$\sigma_{t}ht\cot\varphi =\sigma_{c}ht\cos\varphi \cos\varphi$$

(3) $$\sigma_{c}=\sigma_{t}/\cos^{2}\varphi$$

The compressive strength of the ECC layers for the truss model can be calculated with the Equation (3). 

### 4.2. Arch Model

Similar to common concrete, we believe that the ultimate strength of ECC decreases when the material is under the biaxial stress condition of tension and compression. Usually, softening coefficient is used to show the effective strength of concrete. So, the effective strength of ECC can be shown as *vf_c_*. Then, the compressive strength for the Arch Model can be calculated via the Equation (4):

(4) $$\sigma_{a}=vf_{c}-\sigma_{c}\cos\left(\alpha -\theta \right)$$

**Figure 10:**
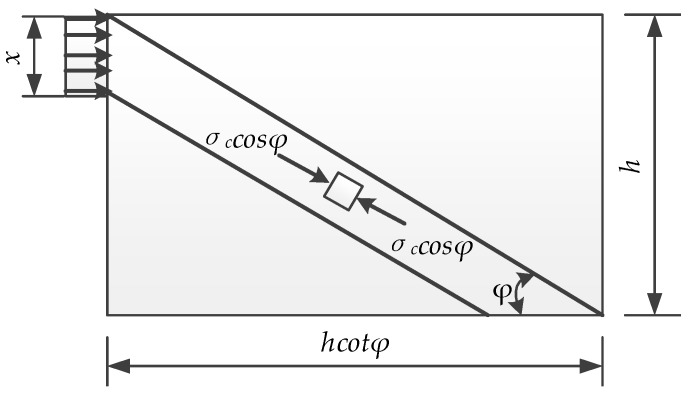
Compressive zone for Truss Model.

In the truss model, *x* represents the vertical width of the concrete compressive zone (as shown in Figure 10). Based on the force balancing condition in the horizontal direction:

(5) $$\sigma_{c}bx=\sigma_{c}ht\cos\varphi \cos\varphi$$

(6) x=hcosφcosφ

**Figure 11:**
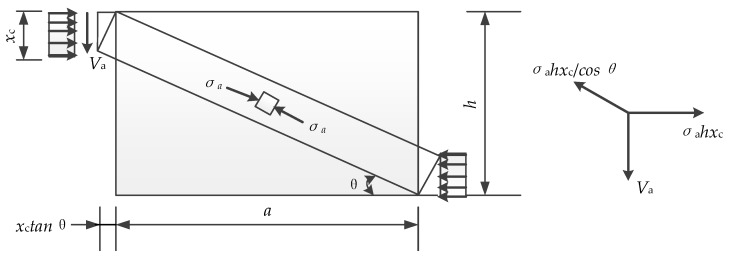
Compressive zone for Arch model.

In the arch model, *x_c_* can be simplified calculated via the Equation (6). So, as shown in Figure 11, the bearing capacity of the arch can be calculated via the Equation (7):

(7) $$V_{a}=\sigma_{a}tx_{c}\tan\theta =\sigma_{a}ht\cos\varphi \cos\varphi \tan\theta$$

From the geometric relations which are shown in Figure 11. It can be calculated that:

(8) $$\tan\theta =\frac{h-x_{c}}{x_{c}\tan\theta +a}$$

(9) $$\tan\theta =\frac{\sqrt{a^{2}+4x_{c}h-4x_{c}{}^{2}}-a}{2x_{c}}$$

The shear bearing capacity of the ECC layer can be calculated by the sum of bearing capacity of the truss and arch. There are two ECC layers for each strengthened beam. Therefore, the increment of shear bearing capacity contributed by the ECC layers can be calculated by the Equation (10):

(10) $$V=2\left(V_{t}+V_{a}\right)=2\left(\eta \beta \sigma_{t}\frac{h}{\cos\varphi }t\cos\varphi +\sigma_{a}ht\cos\varphi \cos\varphi \tan\theta \right)$$

Based on the research conducted before, the Arch model effect takes an important role in the shear behaviors of RC beams only when the shear span-to-depth ratio is under 2.5 [46]. So, in this research, we consider the Arch model effect only for the beams SA-20-2, SB-20-2, SA-40-2, and SB-40-2.

### 4.3. Values of the Correlation Coefficients in These Equations

#### 4.3.1. Reduction Coefficient *η*

Based on the four-point bending experiments, it is easy to understand that with the increase in ECC layers’ thickness, the risk of the deboning failure increase. We suggest that *η* can be simply set to 0.6 and 0.4 for the ECC layer’s thickness are 20 and 40 mm, respectively. 

#### 4.3.2. Influence Coefficient of Reinforcement Ratio *β*

With the strengthening of ECC layers, the tensile reinforcement ratios of the beams changed. As observed from the experiments, reinforcement ratios affect ultimate loads. For that, we use the factor *β* to show the effect of reinforcement ratios on the ultimate loads in the truss model. We suggest that the factor *β* can be calculated by the Equation which is shown below:

*β* = −23.04*ρ* + 1.00 (11)

#### 4.3.3. Angle of the Diagonal Crack

Based on the research conducted before [47] and the failure modes, the diagonal angle was assigned as 45° in this paper. From Equation (1), it is conservative to assign the diagonal angle to that value. 

#### 4.3.4. Softening Coefficient of Concrete

For softening coefficient of concrete, many studies have been conducted. According to Japanese codes, the softening coefficient can be calculated by *ν* = 0.7 − *f_c_*/200 [48]. According to European codes, the softening coefficient can be calculated by *ν* = 0.6 (1 − *f_c_*/250) [49]. And, according to American Concrete Institute codes, softening coefficient is set at 0.6 [50].

For now, research on the softening coefficient of ECC is hard to find. In the research conducted by Shimizu [14] *ν* = 1.7*f_c_* − 0.333 was used [51]. It is known that ECC has ultra-ductile property due to the bridging effect of fibers. In this research, we chose the largest one for common concrete as the softening coefficient for ECC. So, we set *ν* as 0.6.

### 4.4. Results and Discussions

Based on the Equations and coefficients, the shear bearing capacities contributed by the ECC layers can be calculated. The results are shown in Table 4.

When *a*/*d* represents 3, the calculated results agree well with the test results. This means the Truss model is suitable to calculate the shear bearing capacity of the ECC layer. The hypothesis that the tensile behaviors of ECC layers work as tension members of the Truss model is supported. 

When *a*/*d* represents 2, the calculated results are about half of the test results, except the specimen SA-40-2. It is quite conservative. This is because the softening coefficient is too conservative. The compressive strength and fiber bridging property of ECC layers have not been fully considered. For SA-40-2, the calculated result is 172% of the test result. That is because the RC beam was strengthened to be an under-reinforced specimen with 40 mm ECC layers. The failure happened before the material performances of the ECC layers were fully used. 

Overall, the truss and arch model is suitable to calculate the strengthening effect of extended ECC layers. Different from common concrete, which shows brittle failure for tensile and compressive tests, ECC has a good ductility performance and strain hardening behavior due to the fiber bridging effect. Therefore, the strength of ECC after cracking should be taken into consideration for calculating the shear strengthening effect of ECC layers. The tensile behaviors of ECC can be regarded as tension members in the truss model. In the arch model, the softening coefficient which is used for common concrete is too conservative for ECC. The softening coefficient for ECC under biaxial stress conditions of tension and compression needs further study. 

## 5. Conclusions

A series of four-point bending experiments were conducted on control specimens and specimens strengthened with ECC layers varying the thickness of ECC, reinforcement ratio and shear span-to-depth ratio. The truss and arch model was used to evaluate the strengthening effects. Conclusions can be drawn as follows:

1. Shear strengthening of RC beams with side ECC layers is effective. The shear bearing capacity of the strengthened specimen can be improved by 89% over the control one.
2. The ECC thickness should be eluded to override the risk of debonding failure of the concrete interface.
3. The reinforcement ratio after strengthening treatment affects the shear bearing capacity. Strengthening an RC beam into an under-reinforced beam should be avoided.
4. The truss and arch model is suitable for calculating the improvement of shear bearing capacity. Based on the truss and arch model, an evaluation of the shear strengthening effect of the extended ECC layers was established. It shows good agreement with experiments results and is conservative.
5. Shear span-to-depth ratio affects the shear bearing capacity. When the shear span-to-depth ratio is 2, both truss and arch effects contribute to the increase of shear bearing capacity. When the shear span-to-depth ratio is 3, one can believe that only the truss effect contributes to that.
6. The tensile behaviors of ECC can be taken into consideration as the tension members in Truss model when calculating the bearing capacity of the specimen. In the arch model, the softening coefficient used for the common concrete is conservative for ECC. The softening coefficient for ECC under biaxial stress conditions of tension and compression needs further study.

## Figures and Tables

**Table 1:** Materials properties.

Type	Dia. (mm)	Net Area (mm^2^)	*f_c_* (MPa)	*f_t_* (MPa)	*f_y_* (MPa)	*ε_y_*	*f_u_* (MPa)	*ε_u_*	*E* (GPa)
Concrete	-	-	32	2.6	-	-	-	-	30.0
ECC	-	-	56	4.0	-	-	-	-	24.9
Rebar	10	50.2655	-	-	259	0.00122	329	0.012	212.3
	16	201.0619	-	-	386	0.00189	478	0.013	203.8
	25	490.8738	-	-	374	0.00186	439	0.011	201.6

**Table 2:** Beams details.

Specimen ID	*L* (mm)	*l* (mm)	*b* × *h* (mm × mm)	*ρ* (%)	*l_l_* (mm)	*t* (mm)	*a*/*d*
CA-2	2100	1800	150 × 300	0.89	772	0	2
CA-3	2100	1800	150 × 300	0.89	258	0	3
CB-2	2100	1800	150 × 300	2.18	790	0	2
CB-3	2100	1800	150 × 300	2.18	285	0	3
SA-20-2	2100	1800	① 150 × 300 ② 190 × 300	① 0.89 ② 0.71	772	20	2
SA-20-3	2100	1800	① 150 × 300 ② 190 × 300	① 0.89 ② 0.71	258	20	3
SB-20-2	2100	1800	① 150 × 300 ② 190 × 300	① 2.18 ② 1.72	790	20	2
SB-20-3	2100	1800	① 150 × 300 ② 190 × 300	① 2.18 ② 1.72	285	20	3
SA-40-2	2100	1800	① 150 × 300 ② 230 × 300	① 0.89 ② 0.58	772	40	2
SA-40-3	2100	1800	① 150 × 300 ② 230 × 300	① 0.89 ② 0.58	258	40	3
SB-40-2	2100	1800	① 150 × 300 ② 230 × 300	① 2.18 ② 1.42	790	40	2
SB-40-3	2100	1800	① 150 × 300 ② 230 × 300	① 2.18 ② 1.42	285	40	3

① data for specimen before strengthening, and ② data for specimen after strengthening.

**Table 3:** Four-point bending experimental results.

Specimen ID	*P_u_* (kN)	*y* (mm)	Δ*P_u_* (kN)	*S* (%)	*τ* (MPa)
CA-2	136	8.51	-	-	1.44
CA-3	85	6.68	-	-	0.90
CB-2	185	9.60	-	-	1.83
CB-3	125	7.09	-	-	1.23
SA-20-2	250	7.44	114	84	2.18
SA-20-3	117	8.60	32	38	1.02
SB-20-2	290	8.38	105	57	2.39
SB-20-3	150	4.53	25	20	1.24
SA-40-2	190	9.18	54	40	1.41
SA-40-3	120	9.86	35	41	0.89
SB-40-2	350	9.14	165	89	2.48
SB-40-3	151	6.30	26	21	1.06

**Table 4:** Experimental and calculated results.

Specimen ID	Experimental (kN)	Calculated (kN)	Calculated/Experimental (%)
SA-20-2	114	54	47
SA-20-3	32	24	75
SB-20-2	105	47	45
SB-20-3	25	17	68
SA-40-2	54	93	172
SA-40-3	35	33	94
SB-40-2	165	86	52
SB-40-3	26	26	100
